# Waste treatment innovation for infusion bottles using soil solution

**DOI:** 10.1371/journal.pone.0273394

**Published:** 2022-08-22

**Authors:** Marsum Marsum, Sunarto Sunarto, Widodo Widodo, Khayan Khayan, Slamet Wardoyo

**Affiliations:** 1 Poltekkes Kemenkes Semarang, Semarang, Indonesia; 2 Poltekkes Kemenkes Banten, Serang, Indonesia; 3 Poltekkes Kemenkes Surabaya, Surabaya, Indonesia; King Saud University, SAUDI ARABIA

## Abstract

The amount of medical waste, especially infusion bottles, is a problem for environmental pollution. Improper management of infusion bottle waste can have an impact on disease transmission. The medical waste treatment used high technology and high costs will be a financial burden, so simple and effective treatment innovations is needed. This study uses an experimental method of removing bacteria from infusion bottles using a mixture of water and Andoso soil as a solution for washing infusion bottle waste. The soil solution concentration used in washing was 45% with a contact time of 2 minutes. The experiment was carried out with two repetitions. The treatment effect on decreasing the number of bacteria using a multiple linear regression mathematical model. The results showed that the disinfection process of bacterial-contaminated infusion bottles using water required rinsing up to six times, whereas using 45% andosol soil solution only rinsed once. The effectiveness of the disinfection of infusion bottles contaminated with bacteria using soil solution reduces the number of bacteria by 98%.

## 1. Introduction

Medical waste is still a problem in every country whose number increases every year. The tremendous potential risk of medical waste on biological disease transmission is a problem in preventing disease transmission [1, 2]. Internationally, medical waste is considered a top and fatal hazard. Improper management of medical waste will impact water, soil, and air pollution and can be a source of direct danger to humans. Treatment of medical or clinical waste requires sophisticated technology and very high treatment costs [3]. Medical waste treatment requires innovation in medical waste treatment to be easily applied in health care facilities [4].

Incineration of medical waste became the preferred method and became the treatment of choice in late 2005. Small incineration facilities that are not equipped with air pollution control traps are no longer operational in certain countries due to regulations on toxic air emissions (e.g., dioxins and furans) [5]. Large-scale medical waste incinerators for the treatment of medical waste generated by most health facilities are widely used in several countries. There is a significant potential risk of developing toxic pollutants in the air from these incinerators if operated and managed improperly, as medical waste usually contains various plastic materials such as polyvinyl chloride (PVC) [6]. Controlling toxic air emissions from medical waste incinerators is a significant challenge in controlling environmental pollution, so it is necessary to look for alternatives to treat medical waste other than incineration.

Infusion bottles are one type of medical waste mainly produced from health care facilities such as hospitals. Infusion bottles can be reused as raw materials for other products after sterilization. The infusion bottle waste treatment process refers to regulations issued by the Indonesian government regarding hospital environmental health, including ecological health requirements for decontamination through sterilization [7]. Disinfection is a process that reduces or eliminates the number of disease-causing pathogenic microorganisms (excluding spores) by physical and chemical means. Some of the disinfection equipment that has been used include autoclave, chemical disinfection, hydroclave, and dry thermal treatment. The use of disinfection with this method is still found in many autoclaves having operational problems in the pre-vacuum, air leakage, inadequate steam penetration into the effluent, and vacuum pumps. Biological indicators still show positive samples [8]. Sterilization is a process that removes all microorganisms by physical and chemical means; One of the requirements for disinfection is that the germ density at the end of the disinfection process is 0–5 CFU/cm2 [7].

Previous research has been able to reduce the number of pathogenic bacteria *Bacillus cereus* contained in syringe solid waste by using several techniques, including autoclave sterilization with a temperature of 121°C for 15 minutes, 45% andosol soil, or with a combination of the two methods to increase potency sterilization of medical waste has proven to be effective [9]. The antimicrobial activity of clay nanocomposites tested against bacterial strains of *S*. *aureus*, Enterobacter faecalis, Pseudomonas aeruginosa, and *E*. *coli* showed that the antibacterial activity depends on pH and the clay matrix [10]. The role of positive divalent and trivalent cations, including iron and aluminum, in the inhibition of bacteria with low pH [11].

## 2. Materials and methods

### 2.1 Sample collection site description and kind of sample collected

The infusion bottle used as the sample came from the Tugurejo Hospital, Semarang. The infusion bottle is collected in a sealed and sealed initial reservoir. Delivery of infusion bottle waste to the laboratory uses a sample box to prevent contamination and maintain sample quality. The test was carried out at the Laboratory of Poltekkes Kemenkes Semarang.

### 2.2 Research design and sample processing

This study uses an experimental design using a Completely Randomized Design. The treatment was focused on the effectiveness of rinsing water and soil to determine the decrease in the number of bacteria in pre-and-post treatment infusion bottle waste. The treatment was carried out by rinsing with sterile Aquades seven times, one of which was using 45% andosol soil with a contact time of 2 min. The treatment was carried out by rinsing with sterile Aquades seven times, one of which was using 45% andosol soil with a contact time of 2 min. The number of repetitions in the treatment in this study was twice. The treatment was implemented by swabs evenly on the infusion bottle area of 5 cm × 5 cm, bacterial culture using the spread plate method, reporting results in units of CFU/cm2. The effect of treatment on decreasing the number of bacteria used a mathematical model of multiple linear regression [12, 13].

### 2.3 Data analysis

To prove that in this study the number of bacteria in all treatment groups in homogeneous or comparable conditions, a one-way analysis of variance (ANOVA) was carried out. To prove the effect of treatment on the number of bacteria using the T-Test test by comparing the decrease in the number of bacteria in the control and treatment groups.

## 3. Results and discussion

### 3.1 Homogeneity of the number of bacteria according to the treatment group

Table 1 illustrates that samples of infusion bottle waste in hospitals were contaminated with bacteria with homogeneous or comparable amounts for all treatment groups (p = 0.571). This situation provides information that researchers looking at the effect of treatment on the number of bacteria do not need to control the number of bacteria before using multivariate analysis.

**Table 1:** Baseline of bacterial count data (CFU/ml^2^) on infusion bottles.

		n	Mean	SD	p*
**Rinsing using soil**	flushing 1	2	22.0	2.83	0.571
	flushing 2	2	22.0	2.83	
	flushing 3	2	22.0	2.83	
	flushing 4	2	22.0	2.83	
	flushing 5	2	22.0	2.83	
	flushing 6	2	22.0	2.83	
	flushing 7	2	22.0	2.83	
	Control	2	22.0	2.83	

*One Way Anova.

### 3.2 Decreased bacterial count due to frequency of rinsing with water

The results of this study indicate that the infusion bottle is contaminated with bacteria when it is only washed with water at a frequency of many times, as shown in Table 2.

**Table 2:** Decrease in the number of bacteria due to the frequency of rinsing with water.

Treatment with a mixture of water and soil	n	Number of bacterial colonies (CFU/ml^2^)	
		Mean	SD
**Before rinsed**	16	22.0	2.83
**First rinse**	16	13.7	1.89
**Second rinse**	16	7.0	1.41
**Third rinse**	16	4.5	1.41
**Fourth rinse**	16	1.3	0.94
**Fifth rinse**	16	0.5	0.71
**Sixth rinse**	16	0.0	0.00
**Seventh rinse**	16	0.0	0.00

Table 2 shows that to clean hospital infusion bottles from bacterial contamination, using water requires rinsing up to 6 times, while rinsing with water and soil only requires rinsing once.

Healthcare waste consists of waste generated by health facilities, medical laboratories, and biomedical research facilities. Improper treatment of this waste poses a severe risk of disease transmission to waste workers, health workers, patients, and the public through exposure to infectious agents. Poor waste management produces hazardous and infectious contaminants in communities. Contamination of highly infectious agents such as the COVID-19 virus has created enormous instability in the handling of healthcare and recycling waste due to the volume of waste generated and its contagious nature.

Proper management of HCW can add value by reducing the spread of the COVID-19 virus and increasing the recycling of materials, rather than sending them to landfills. Disinfection and segregation of health care waste facilitate sustainable management and enable its use for valuable purposes [14].

The amount of medical waste that causes complex problems is associated with high processing costs. Legislation requires medical or clinical waste to be processed to avoid nosocomial and other environmental pollution. The treatment of medical or clinical waste requires more technology and has very high processing costs. Medical waste treatment requires innovation in processing medical waste that is easy to apply in healthcare facilities [4].

Natural clay and rice husk modified with Na_2_CO_3_ were used to produce clay aggregate adsorbents for the disinfection of *E*. *coli* in water [15]. The use of clay and its PCH material can be a suitable method for removing Salmonella from water. The large adsorption capacity of Salmonella started from the lowest value in the mont-PCH sample (0.29 × 1010 CFU g −1) to the highest value in the natural palygorskite sample (1.52 × 1010 CFU g − 1) [16].

### 3.3 Effect of soil solution on reducing bacterial count

The ability of the soil, both physically and chemically, causes bacterial death. One of the mechanisms by which soil kills bacteria is the use of the minerals present in it. Minerals contained in clay such as silicate tetrahedral (SiO_4_) and octahedra (consisting of Al, Mg, and Fe) are building blocks of minerals that can exchange cations and release metal cations that encourage antibacterial reactions [17]. Mineral natural clay has antibacterial properties, one of which is based on reduced iron (Fe^2+^). Fe^2+^ ions can enter the protein structure of bacterial outer cells and produce hydroxyl radicals that kill bacteria [18]. The presence of other transition metals and Al^3+^ carried by natural clay also plays a direct role in this antibacterial property [19]. Clay minerals also act as a support for metals which are known to be very active as antibacterials [20] Soil can absorb ions that are close to the active soil material (miscellaneous). The sorption ability decreased as the radius increased. In other words, the more concentrated the concentration of the soil solution, the tighter the space, and the larger the sorption capacity. This binding/absorbing ability decreases with low water content; this is due to the ability of the micelles to be properly active when the soil is in a solution or aqueous condition [21].

**Fig 1:**
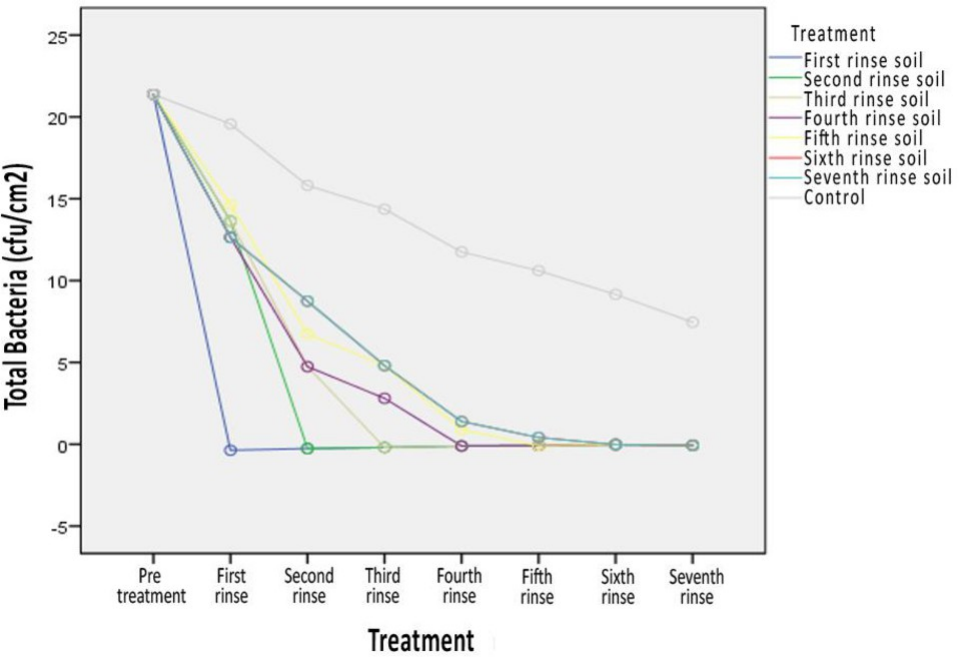
Changes in the decrease in the number of bacteria between the treatment group and the control group.

Cleaning or reducing the number of bacteria in the infusion bottle waste is a reasonable effort before the process is utilized in other products. In this study, the infusion bottle waste was repeatedly rinsed with various treatments. The effect of decreasing the number of bacteria varies according to the type of treatment given, as shown in Fig 1.

Fig 1 shows that initially, all treatment groups had the same number of bacteria, which was 21.4; after administration of various treatments, the position of groundwater rinsed, in any position when rinsed with groundwater, was able to clean the bacterial contamination of the infusion bottle waste. The decrease in the number of bacterial effects from the treatment after being analyzed using a linear regression mathematical model, which was controlled through analysis by the number of pre-bacteria, resulted in a number of differences in the number of bacteria for each treatment compared to the control group, as shown by the regression coefficient (B), in Table 3.

**Table 3:** Effect of flushing treatment of water and soil mixture at various positions on the decrease in bacterial count.

	B	SE	T	p*	95% Confidence Interval		Partial Eta Squared
Treatment with a mixture of groundwater					Lower Bound	Upper Bound	
First rinse	-19.9	1.1	-17.9	<0.001	-22.6	-17.3	98%
Second rinse	-16.1	1.3	-12.5	<0.001	-19.1	-13.0	96%
Third rinse	-14.6	0.9	-15.3	<0.001	-16.8	-12.3	97%
Fourth rinse	-11.9	0.8	-14.5	<0.001	-13.8	-9.9	97%
Fifth rinse	-10.7	1.3	-8.4	<0.001	-13.7	-7.7	91%
Sixth rinse	-9.2	0.6	-14.6	<0.001	-10.7	-7.7	97%
Seventh rinse	-7.5	1.9	-4.0	0.005	-12.0	-3.1	69%
Control							

*T-Tes; Significant ≥0.005.

Based on Table 3, of the seven positions of rinsing groundwater, starting from groundwater to rinse infusion bottles contaminated with bacteria, the first rinse directly with groundwater to rinse groundwater at the seventh rinse position can reduce the number of different bacteria and is increasingly in the backward position. The ability to reduce the number of bacteria decreased. However, in any position, treatment when rinsed with ground water was able to clean the bacteria that contaminate the infusion bottle. As stated in the table, the infusion bottle contaminated with bacteria when directly washed with groundwater was able to reduce the number of bacteria by 19.9 CFU lower than the control group, and the decrease was statistically significant (p< 0.001). The first rinse with groundwater was able to clean up 98% of bacteria. In subsequent rinses from the second to seventh treatments, the reduction of bacteria appeared to be less effective than if the water and soil treatments were carried out in the first rinse. However, treatment in the first to seventh rinses was still proven to reduce the number of bacteria. This difference in effectiveness may be due to differences in the ability and viability of different bacteria found on the plabot. Therefore, there is a possibility that bacteria that survive in the final rinse are more resistant to the antibacterial activity of the soil. Other studies have suggested that soil and mineral content in modified soils can be used to remove pathogenic bacteria. The mechanisms that occur in this process are mainly cation exchange and ion-dipole coordination interactions and hydrogen bonds, which largely depend on the isoelectric point, the size of the molecule, the shape of the molecule, and the pH of the solution [22]. Clay naturally contains minerals that can be dissolved in water. Soil pH with hydrated antibacterial properties was generally high (> 10) or low (< 5), where soluble Al and Fe were found. The presence of Fe and Al compounds and other transition metals has a direct role in the antibacterial properties of the soil [23, 24].

Research by Morrison et al. [23] also showed that soil kills pathogenic compounds within < 24 h with chemical toxicity that occurs, and not physically. When antibacterial soil removed from its natural environment and activated with deionized water for medical applications, soil rebalances with new fluids. During this process, dissolved and oxidized minerals such as pyrite, plagioclase, and smectite release metals that suppress the growth of pathogenic bacteria.

Mixing soil with water produces a mixture of solutions, colloids, and suspensions of clay and humus. Humus particles dissolved in water produce a solution that can function as a surfactant. The combination of clay nanoparticles and surfactants is known to have antiviral properties [25] so it can be understood that the presence of water and soil produces a system that contains antiviral powers. The antibacterial properties are also attributed to the clay from dissolved aluminum [26].

To see the difference in the effects of the seven types of treatment after being tested using the least significant difference (LSD), which is indicated by the difference in mean between groups, as shown in Table 4.

**Table 4:** Differences in the decrease in the number of bacteria between treatments.

Treatments		Mean difference	SE	p	95% Confidence Interval For Difference	
					Lower Bound	Upper Bound
**First rinse soil**	Second rinse soil	-1.75	0.67	0.035	-3.34	-0.16
	Third rinse soil	-2.38	0.67	0.010	-3.97	-0.79
	Fourth rinse soil	-2.63	0.67	0.006	-4.22	-1.04
	Fifth rinse soil	-3.50	0.67	0.001	-5.09	-1.91
	Sixth rinse soil	-3.63	0.67	0.001	-5.22	-2.04
	Seventh rinse soil	-3.63	0.67	0.001	-5.22	-2.04
	Control	-11.22	0.81	0.000	-13.13	-9.32
**Second rinse soil**	Third rinse soil	-0.63	0.67	0.0384	-2.22	0.97
	Fourth rinse soil	-0.88	0.67	0.234	-2.47	0.72
	Fifth rinse soil	-1.75	0.67	0.035	-3.34	-0.16
	Sixth rinse soil	-1.88	0.67	0.027	-3.47	-0.29
	Seventh rinse soil	-1.88	0.67	0.027	-3.47	-0.29
	Control	-9.47	0.81	0.000	-11.38	-7.57
**Third rinse soil**	Fourth rinse soil	-0.25	0.67	0.721	-1.84	1.34
	Fifth rinse soil	-1.13	0.67	0.138	-2.72	0.47
	Sixth rinse soil	-1.25	0.67	0.105	-2.84	0.34
	Seventh rinse soil	-1.25	0.67	0.105	-2.84	0.34
	Control	-8.85	0.81	0.000	-10.75	-6.95
**Fourth rinse soil**	Fifth rinse soil	-0.88	0.67	0.234	-2.47	0.72
	Sixth rinse soil	-1.00	0.67	0.181	-2.59	0.59
	Seventh rinse soil	-1.00	0.67	0.181	-2.59	0.59
	Control	-8.599	0.80	0.000	-10.50	-6.70
**Fifth rinse soil**	Sixth rinse soil	-0.13	0.67	0.858	-1.72	1.47
	Seventh rinse soil	-0.13	0.67	0.858	-1.72	1.47
	Control	-7.72	0.81	0.000	-9.63	-5.82
**Sixth rinse soil**	Seventh rinse soil	-0.00	0.67	1.000	-1.59	-1.59
	Control	-7.60	0.81	0.000	-9.50	-5.70
**Seventh rinse soil**	Control	-7.60	0.81	0.000	-9.50	-5.70

Table 4 illustrates that rinsing the infusion bottle contaminated with bacteria directly with groundwater is better than rinsing in the second position (p = 0.035). The difference in the decrease in the number of bacteria was 11.2 compared to the control group.

As a health service, the hospital provides inpatient and outpatient services and produces medical waste, which forms part of a group of hazardous and toxic wastes. Maintenance of the hospital environment is regulated by the Regulation of the Minister of Health of the Republic of Indonesia No. 7 of 2019 concerning. The handling of hazardous and toxic waste is regulated by government regulation No. 22 of 2021 concerning environmental management. This study is committed to managing B3 waste, especially in infusion bottles, so that they may be reprocessed. One of the efforts is to reduce or eliminate bacteria that contaminate infusion bottles [7, 27].

Maharani et al. [28] grouped medical hazardous and toxic waste at the Soedirman Semarang Hospital, including syringes, disposable masks, disposable gloves, infusion bottles, scalpels, contaminated cotton, contaminated gauze, medicine bottles, and scalpel infusion hoses [28].

Incineration is the technology used to remove hazardous waste. The incineration system can reduce the volume of medical waste by up to 99.95%. In addition, incineration technology can digest medical waste materials that are harmful to the environment and destroy pathogenic bacteria. However, the incineration system has drawbacks. One disadvantage is that it produces residual ash and waste that still contain heavy metals. Therefore, improvements in incineration management are required. One of the efforts that can be made is the treatment of residues and ash waste [29].

According to Marsum et al. [30], waste treatment before use can adopt several techniques, including autoclave sterilization at a temperature of 121°C for 15 min, 45% andosol soil with a contact time of 2 min, or a combination of the two methods to increase the sterilization power of medical waste [30]. Andosol, which is very abundant, can be used as an anti-bacterial by applying tubular SiO2-NT and Cu nanocomposites that can inhibit the growth of *E*. *coli* and *S*. *aureus* effectively with minimal inhibitory concentrations (MIC) of 2.0 mg/mL and 0.6 mg/mL [31].

Antimicrobial activity of andosol nanocomposites against bacterial strains of *S*. *aureus*, *E*. *faecalis*, *P*. *aeruginosa*, and *E*. *coli*, was evaluated by determining the minimum inhibitory concentration. The antibacterial activity was found to depend on the pH and the clay matrix [10]. Role of divalent and trivalent positive cations including iron and aluminum in the inhibition of bacteria at low pH [11].

## 4. Conclusion

Treatment of medical waste in infusion bottles can be carried out using the sterilization method using soil as a disinfectant. Using a mixture of soil and water has better effectiveness than washing infusion bottles without using soil. Statistically, the bacterial removal of infusion bottles using water and soil by washing with water had a significant difference in the number of bacteria in each washing step. The effectiveness of the disinfection of the water and soil mixture reaches 98% in the first rinse or wash. The research can be continued with the treatment of various types of soil, both sterilized and soil in natural conditions.

## Supporting information

S1 File (PDF)
