# Supplementary material for: Waste treatment innovation for infusion bottles using soil solution
Source: PLoS One. 2022 Aug 22;17(8):e0273394. doi: 10.1371/journal.pone.0273394 (PMC9394799; doi:10.1371/journal.pone.0273394)
Supplement: S1 File — (PDF) [file pone.0273394.s001.pdf]

Sample code	Treatment	Before rinsing	Number of Bacteria_1	Number of Bacteria_2	Number of Bacteria_3	Number of Bacteria_4	Number of Bacteria_5	Number of Bacteria_6	Number of Bacteria_7
A1	First rinse soil	20	0	0	0	0	0	0	0
A2	First rinse soil	24	0	0	0	0	0	0	0
B1	Second rinse soil	20	12	0	0	0	0	0	0
B2	Second rinse soil	24	16	0	0	0	0	0	0
C1	Third rinse soil	20	12	4	0	0	0	0	0
C2	Third rinse soil	24	16	6	0	0	0	0	0
D1	Fourth rinse soil	20	12	4	2	0	0	0	0
D2	Fourth rinse soil	24	14	6	4	0	0	0	0
E1	Fifth rinse soil	20	14	6	4	0	0	0	0
E2	Fifth rinse soil	24	16	8	6	2	0	0	0
F1	Sixth rinse soil	20	12	8	4	1	0	0	0
F2	Sixth rinse soil	24	14	10	6	2	1	0	0
G1	Seventh rinse soil	20	12	8	4	1	0	0	0
G2	Seventh rinse soil	24	14	10	6	2	1	0	0
H1	Control	20	20	20	20	20	20	20	20
H2	Control	24	24	24	24	24	24	24	24
